# Strain-enhanced Dzyaloshinskii–Moriya interaction at Co/Pt interfaces

**DOI:** 10.1038/s41598-020-69360-w

**Published:** 2020-07-23

**Authors:** Caner Deger

**Affiliations:** Department of Physics, Marmara University, 34722 Ziverbey, Istanbul, Turkey

**Keywords:** Magnetic properties and materials, Spintronics

## Abstract

The interfacial Dzyaloshinskii–Moriya interaction (DMI) is an essential ingredient for stabilizing chiral spin configurations in spintronic applications. Here, via first-principles calculations, we reveal the influence of lattice strain on DMI in Co/Pt interface. We observed a considerable enhancement for a certain lattice strain. Furthermore, a direct correlation is established between the DMI and interlayer distances dominated by the strain, which is attributed to a hybridization of electronic orbitals. This hybridization has also been presented as the microscopic origin of the interfacial DMI. We anticipate that our predictions provide new insights into the control of interfacial DMI for skyrmion-based spintronic devices.

## Introduction

Chiral magnetic structures have attracted the interest of numerous research owing to the diversity of promising spintronic devices^1–6^. The adjustment of chiral spin textures to cutting-edge memory and logic technology can pave the way for the fabrication of compact sized and energy-efficient devices with high magnetic stability. Broken inversion symmetry in magnetic materials can induce the formation of chiral spin textures. Especially, ferromagnetic/heavy metal multilayers with broken inversion symmetry have been employed to obtain room-temperature zero-field skyrmions^7–9^. Such interfaces can stimulate strong Dzyaloshinskii–Moriya interaction (DMI)^10–13^ which stabilizes noncollinear chiral magnetic structures. Tailoring magnetic topography of these structures requires both a deep understanding and a precise control of the DMI. A large number of studies have been carried out to understand the physical mechanisms underlying the interaction^14–16^. It is generally reported that electronic configuration of materials constituting the interface, designates the DMI^17–20^. The correlations between 5*d* band filling, Hund’s first rule and DMI are recently revealed^20,21^.

Understanding of the mechanisms are followed by the investigations on controlling the sign and strength of the DMI which determines the direction of motion of chiral spin textures, e.g. skyrmions and domain walls, driven by an electric current via spin–orbit torques^3,22–24^. Previous investigations aiming to maximize DMI have focused on three different approach: (1) altering the thickness of the magnetic and/or non-magnetic layers in which gathered to form interfacial DMI. Varying ferromagnetic layer thickness generally influences the number of magnetic dead layer and transverse spin diffusion length^25,26^, while the thickness of non-magnetic layer affects the cumulative electron hopping between the atomic spins at the interface and the non-magnetic atoms in the non-magnetic metal layer^27^. (2) changing stacking order or material, which can naturally vanish the DMI for the FM layer sandwiched by the same HM layers due to the inversion symmetry, to enhance total DMI in the stack^14, 15, 28^ (3) designing electronic band structure and charge carrier density by varying the chemical composition of both magnetic and non-magnetic layers^29,30^. Other internal or external effects inducing the DMI are also investigated in the literature^16,31,32^. In addition to the methods mentioned above, an unsophisticated phenomenon occuring at interfaces can lead to enhanced DMI: crystal lattice strain. Effect of the strain on the DMI is previously reported for bulk metallic helimagnets^33^ and bcc Fe crystal with strain gradient^34^. However, the influence of the strain on interfacial DMI, which is essential to stabilize the Néel type spin configuration in magnetic skyrmions and domain walls with certain chirality, has not been reported yet. A lattice strain at ferromagnetic metal (FM)/heavy metal (HM) interfaces provided by a suitable substrate or buffer layer, can influence the DMI.

In this paper, we reveal the behaviour of DMI at FM/HM interfaces as a function of lattice strain from first principles calculations. To characterize DMI, we choose a well-studied system; Co/Pt interface^14,35–38^. We observed that a slight variation of lattice strain leads to a significant enhancement in DMI, which is evaluated quantitatively by measuring the self-consistent total energy of the systems with opposite chirality. Chirality-dependent energy difference of individual layers ($\Delta E_{CW-CCW}$) are also calculated to uncover the dependence of the interaction on the distance between layers. To comprehend the enhancement of the interaction at a specific strain, we associated the interaction with occupied and unoccupied energy eigenvalues at the $\Gamma$ point relative to the Fermi energy. We expect that our study not only offers a rational design for controlling the strength of the DMI but also will inspire future considerations about the influence of lattice strain on chiral spin textures originated by interfacial Dzyaloshinskii–Moriya interaction.

## Methods

DMI calculations are performed by constrained spin method implemented in Vienna ab initio simulation package (VASP)^39,40^, which is previously employed for DMI calculations in bulk frustrated systems^41^, insulating chiral-lattice magnets^42^ and finally FM/HM interfaces^14,15,28,43^. Generalized gradient approximation (GGA) of Perdew–Burke–Ernzerhof (PBE) type functional for electron–electron interactions and projector augmented wave method (PAW) for electron-ion interactions^44^ are used for the calculations with the cut-off energies for the plane-wave basis sets of 400 eV. A supercell consisting of 2 atomic monolayers (ML) of Co on 2 ML of Pt is employed to form Co(2)/Pt(2) structure. The z component of the DMI vector is calculated using opposite spin chirality configurations, where the supercell contains a row of 4 atoms in the plane. Thus, the cycloid wavelength is chosen as $n=4$ for convenience. A 6 ML-thick vacuum slab was considered along the thickness direction to avoid the interaction between the repeating slabs. $\Gamma$-centered 4 $\times$ 16 $\times$ 1 k-point sampling, which is adequate to reach the convergence, is employed for the Brillouin-zone mesh. A structural relaxation is performed to obtain most stable geometry until the forces become smaller than 0.001 eV/Å. Following the geometry optimization, we fixed the in-plane lattice constant, a, and varied the out-of-plane lattice constant, c, of the whole crystal in order to apply the strain. Thus, both Pt and Co layers are subjected to this strain, which can be experimentally achieved by a proper substrate or a buffer layer. Electronic charge distribution of the structure is then calculated by solving Kohn–Sham equations in the absence of spin–orbit coupling. Finally, clockwise (CW) and counter-clockwise (CCW) spin spirals are constructed across the supercell using the constrained spin method^14^. The total DMI strength, $d^{tot}$ is evaluated based on the total energies for CW and CCW spin spiral configurations assuming coherent rotation of the spin moments in all layers of the supercell. The self-consistent total energy difference between these two configurations was used to compute the DMI in the presence of spin–orbit coupling and scaled by the geometry as^14^

1 $$\begin{aligned} d^{tot}=\frac{E_{CW}-{E_{CCW}}}{n sin\left( \frac{2\pi }{n} \right) }. \end{aligned}$$

This relation considers nearest-neighbor exchange interaction, i.e. $\vec{D}_{ij} \ne 0$ only if sites *i* and *j* are nearest-neighbors. Chirality-dependent energy difference of individual layers, $\Delta E_{CW-CCW}$, are calculated by considering the energy difference between the spin configurations with opposite chirality in a single layer while spins in all other layers are constrained to be along the *y*-axis.

## Results and discussion

**Figure 1:**
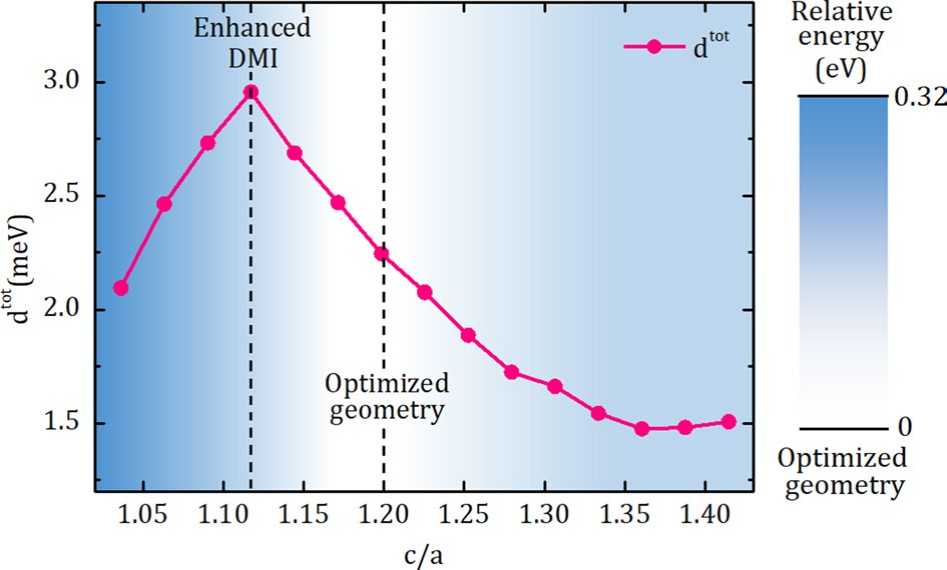
The dependence of the total DMI coefficient $d^{tot}$ on the ratio of lattice parameters; c/a. The background gradient shows the formation energy relative to the optimized geometry marked by the dashed line at $c/a\approx 1.2$. The enhanced-DMI is obtained at $c/a\approx 1.1$. Positive $d^{tot}$ corresponding counter-clockwise chirality is calculated for all ratios.

Total DMI strength, $d^{tot}$, of hcp(0001)Co/fcc(111)Pt interface with different c/a ratio, from 1.05 to 1.40, is given in Fig. 1. Prior to interpreting influence of lattice strain on $d^{tot}$, we should note that blue-gradient in the background of the data represents the calculated relative formation energy with respect to the optimized geometry corresponding the white-backgrounded region. This energy is scaled by the number of unit cells in the above mentioned supercell. For $d^{tot}$, we find a large variation in the range 1.5–3 MeV of CCW chirality. Increasing compressive strain (increasing c/a ratio beyond the optimized geometry) in the Co/Pt interface causes a systematic decreasing trend in $d^{tot}$. However, a tensile strain of 7% at the interface (c/a $\sim$ 1.1) results in a considerable enhancement up to 32% in $d^{tot}$. The enhancement is gradually vanished with increasing tensile strain. This amount of strain can be applied to the Co/Pt interface by suitable substrates, buffer layers or piezoelectric materials. For this purpose, we should consider the interaction between the surface on which to form the thin film and the DMI-active layers of Co/Pt interface. Besides, determining DMI originated from each layer can improve our understanding on the $d^{tot}$ which might be influenced by the distance between layers constituting the interface.

**Figure 2:**
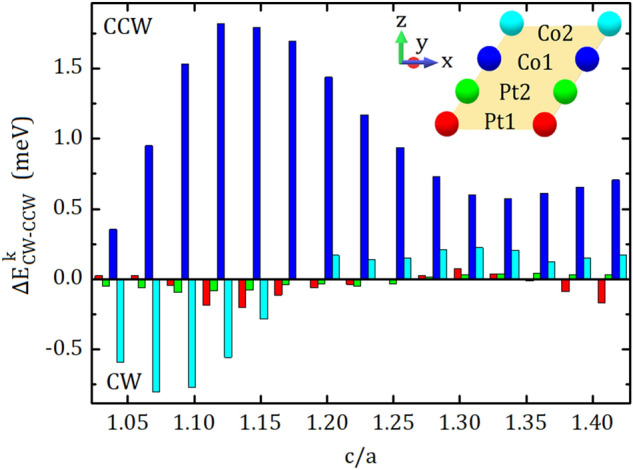
Chirality-dependent energy difference of individual layers, $\Delta E_{CW-CCW}$, as a function of c/a. Red, green, blue and cyan colored bars indicate DMI coefficients of Pt1, Pt2, Co1 and Co2 layers in the schematic representation at the top-right of the figure, respectively.

In order to determine DMI-active layers and their contributions to the $d^{tot}$, we calculate the chirality-dependent energy difference of individual layers ($\Delta E_{CW-CCW}$). The results are shown in Fig. 2. The $\Delta E_{CW-CCW}$ belonging to each layer is represented by the color bars, which encoded to the color of perpendicularly-arranged layers at the top-right of the figure. It is obvious that the interfacial Co layer, named as Co1 and indicated by the blue bar, is predominant in the distribution of $\Delta E_{CW-CCW}$. The preferred chirality of interfacial Co layer (k $=$ Co1) is CCW for all c/a values while that of Co2 layer is changing from CW to CCW with increasing c/a. In other words, Co1 and Co2 layers has the same chirality for larger c/a values, however, decreasing c/a forces their chirality to become opposite. Also, the magnitude of the $\Delta E_{CW-CCW}$ at Co2 is increasing with decreasing c/a. Large DMI between Co spins at Co1 layer can be associated with a large spin–orbit coupling in the adjacent Pt layer. Within this context, the change in the interlayer distances caused by the lattice strain, determines the sign and magnitude of layer resolved DMIs. For smaller c/a values, increasing magnitude of $\Delta E_{CW-CCW}$ at Co2 is caused by the decreasing distance between the Co2 layer and the interface. On the other hand, the DMI of Co2 layer is originated by not only interfacial Pt layer but also adjacent Co1 layer. The hybridization between interfacial Pt and adjacent Co layers is conducted by the change in the interlayer distances caused by the lattice strain, which eventually dominates the sign and magnitude of the DMI. Apart from these, the contributions from both Pt layers to the DMI is negligible.

**Figure 3:**
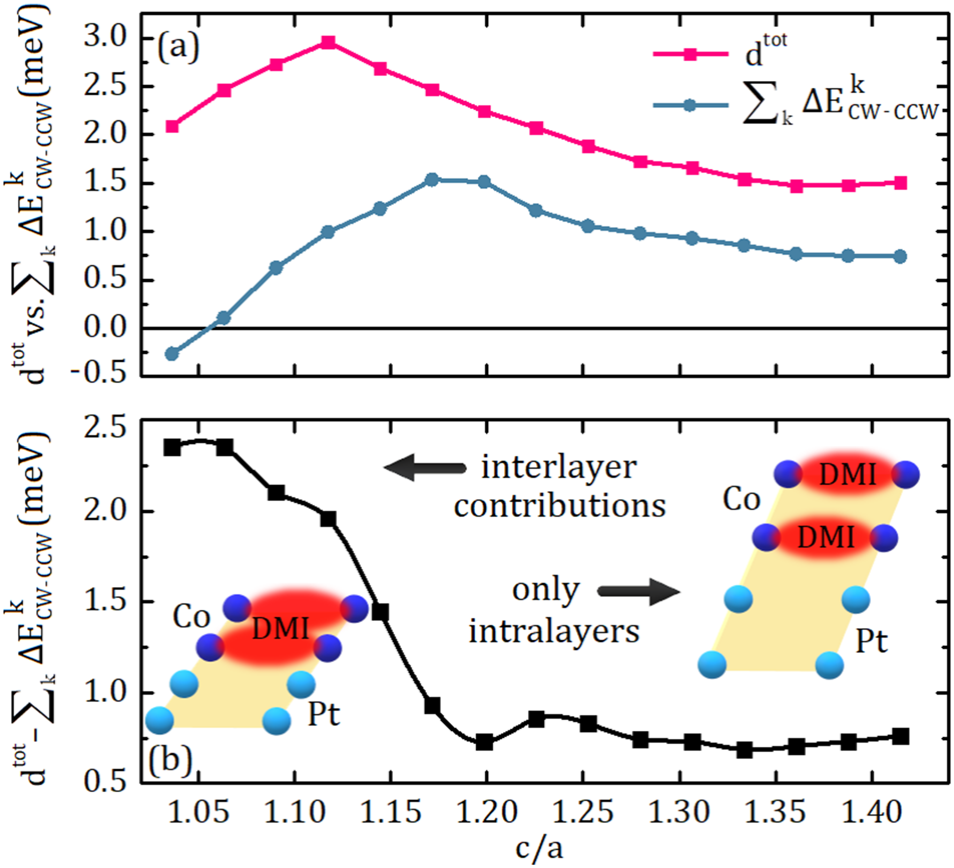
(**a**) The total DMI coefficient, $d^{tot}$ versus the sum of $\Delta E_{CW-CCW}$ of all four layers as a function of c/a. (**b**) The difference occured between $d^{tot}$ and the sum of $\Delta E_{CW-CCW}$. The regions where dominated by either interlayer contributions or intralayer properties are pointed by the arrows. The schematic representations indicate the influence of perpendicular interlayer distances on $d^{tot}$ for smaller and larger c/a ratios.

We compile our discussion on the contributions of individual layers to the DMI by comparing $d^{tot}$ with the sum of $\Delta E_{CW-CCW}$ of different layers as shown in Fig. 3. At first sight, $d^{tot}$ and the sum of $\Delta E_{CW-CCW}$ have a similar trend as a function of c/a in Fig. 3a. Especially, for larger c/a values, the difference is moderately small and it can be described by the fact that DFT calculations on differently constrained spin configurations can not be strictly equivalent^14,28^. However, the large difference for smaller c/a values can not be explained by the nature of DFT calculations and it deserves to be separately discussed. Thus, we generate Fig. 3b by subtracting the sum of $\Delta E_{CW-CCW}$ from $d^{tot}$. It can be expected that in a physical system at equilibrium, spin spirals in neighboring layers are almost in phase due to interlayer exchange energy. This condition is not satisfied when we assume CW in Co1 and CCW in Co2 which results in large increase of exchange energy. The discrepancy between $d^{tot}$ and $\sum ^{k} \Delta E_{CW-CCW}$ in Fig. 3b is attributed to the interlayer contributions which are integrated into $d^{tot}$ but not into the sum of $\Delta E_{CW-CCW}$. Tensile stress in the lattice ($\hbox {c}/\hbox {a}\le 1.2$) give rise to a decrease in the interlayer distances, boosting the interlayer contributions which is not taken into account in the sum of $\Delta E_{CW-CCW}$. Conversely, compressive stress ($\hbox {c}/\hbox {a}\ge 1.2$) increases the distance between layers and allows each layer to behave as an individual layer. In this manner, the sum of the $\Delta E_{CW-CCW}$ is converging to $d^{tot}$ for larger c/a ratios.

**Figure 4:**
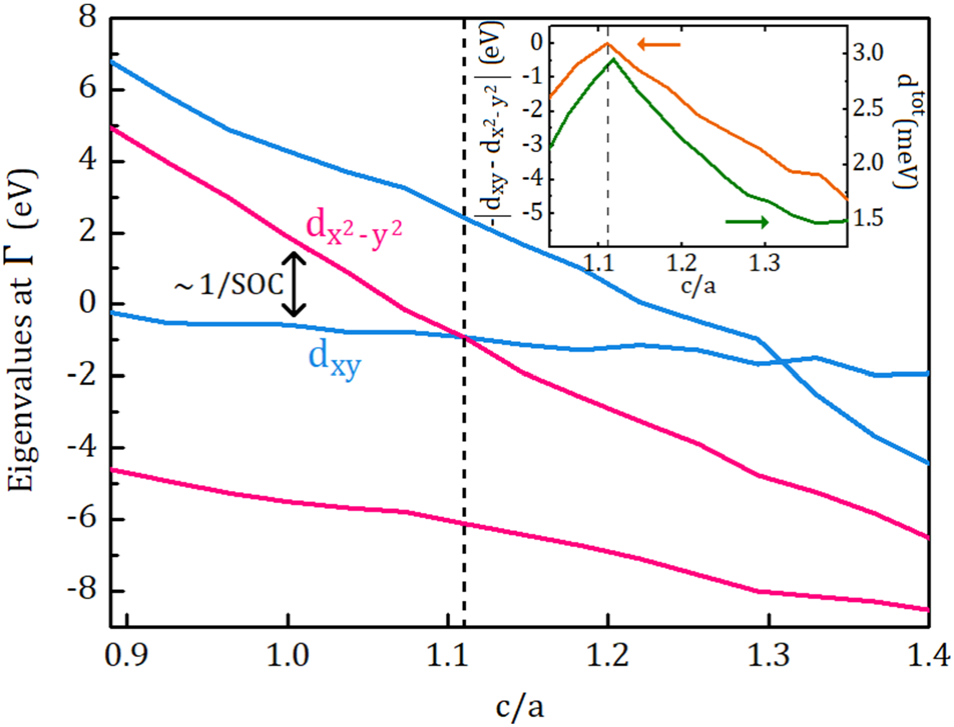
Energy eigenvalues at the $\Gamma$-point relative to the Fermi energy of the system. The pink ($d_{x^2-y^2}$ character) and blue ($d_{xy}$ character) lines closer to the Fermi level are unoccupied and occupied energy levels, respectively. The difference between $d_{x^2-y^2}$ and $d_{xy}$ is associated by the inverse of the spin–orbit coupling (see text). The dashed line indicates the crossing point. The inset shows the relation between the energy difference and $d^{tot}$. We consider the negative absolute value of the energy difference for convenience.

Up to this point, we have discussed the DMI of the layers and their interactions. To clarify the underlying mechanism of the strain-enhanced DMI at the adjacent Pt and Co layers (Pt2 and Co1) dominating the behaviour of the structure, we should look closer to the interface. Although we inferred that the alteration in the distance between Pt and Co atoms contributes to the DMI, further calculations were needed to conceive why specific strain leads to enhanced-DMI. Previously, the effect of strain on magnetic anisotropy energy (MAE), as another quantity related to spin–orbit coupling, was investigated by ab-initio calculations^45^. In their study, orbital hybridization in electronic band structure of strained FeCo alloys was introduced as the source of enhanced MAE. To investigate the effect of strain on the band structure of Co/Pt interface, we employ a supercell consists of two adjacent primitive cell, i.e., one Co atom on top of Pt atom. This simplified interface enabled us to pursue certain energy levels and identify relevant information in the otherwise complex electronic band structure. In Fig. 4, the occupied and unoccupied energy eigenvalues at the $\Gamma$-point relative to the Fermi energy of the system are presented as a function of c/a ratio. Among different energy levels around the orbitally-mixed Fermi level, the levels of which exhibiting predominant $d_{x^2-y^2}$ and $d_{xy}$ character are only considered. For $c/a<1.1$ in Fig. 4, the unoccupied and occupied energy levels are dominated by $d_{x^2-y^2}$ and $d_{xy}$ orbitals, respectively. The energy of $d_{x^2-y^2}$ orbital decreases while that of $d_{xy}$ orbital remains nearly constant with increasing *c*/*a*. The energy levels are inevitably intersected at $c/a\approx 1.1$, arising from the orbital hybridization. As can be seen in the inset of Fig. 4, total DMI of the interface is strictly dependent on the inverse of the energy difference between occupied and unoccupied eigenvalues. The matrix elements of the spin–orbit interaction between occupied and unoccupied states is responsible for MAE according to the second order perturbation theory^45^ while DMI is one of the example of relativistic magnetic interactions appears in the first order of the spin–orbit coupling^46,47^. Thus, the energy difference between occupied and unoccupied states near the Fermi level also plays a key role in determining the strength of the DMI^48^.

**Figure 5:**
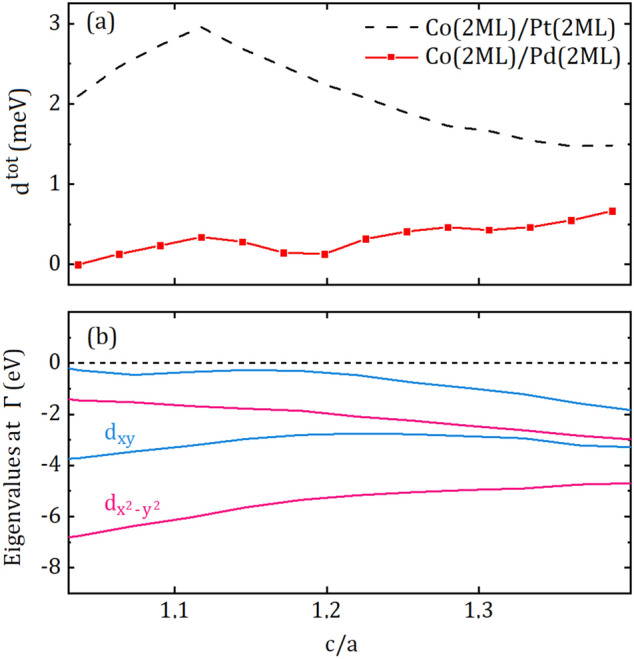
(**a**) The dependence of the total DMI coefficient $d^{tot}$ of Co(2)/Pd(2) on the ratio of lattice parameters; c/a. $d^{tot}$ of Co(2)/Pt(2) (dashed line) is given for comparison. (**b**) Energy eigenvalues at the $\Gamma$-point relative to the Fermi energy of the system. The dashed line indicates the Fermi level of the system.

In order to discuss simultaneously the effects of lattice strain and element substitution on DMI of such systems, we present the total DMI coefficient of Co(2)/Pd(2) system with respect to c/a in Fig. 5a. The relaxed c/a ratio of the Co/Pd system is $c/a\approx 1.2$, which is close to that of Co/Pt, due to the similar lattice parameters of Pd and Pt. We represent the dependency of $d^{tot}$ of Co(2)/Pt(2) system on c/a, reproduced by the same data set in Fig. 1, for comparison. There is an increasing trend for $d^{tot}$ of Co/Pd interface with increasing c/a ratio, however, the size of the $d^{tot}$ is considerable small, when compared to that of Co/Pt. Co/Pd and Co/Pt interfaces are frequently investigated for spin–orbit torque purposes in the literature^49,50^ since both Pd and Pt have similar electronic shell structures and their interface interactions with Co give rise to perpendicular magnetic anisotropy. Thus, the significant difference between their lattice strain dependent DMIs must be investigated. In Fig. 5b, we, again, employ $d_{x^2-y^2}$ and $d_{xy}$ orbitals of Co/Pd interface, as a function of c/a ratio. With increasing c/a ratio, the difference between $d_{x^2-y^2}$ and $d_{xy}$ is decreasing and they are almost intersected at $c/a> 1.3$ while the DMI of the Co/Pd is slightly increasing. However, the DMI of Co/Pd is still 3 times smaller than that of Co/Pt. Element substitution changes not only crystal geometry of the interface but also the position of the Fermi level of the structure. Even if the inverse of the energy difference between $d_{x^2-y^2}$ and $d_{xy}$ is increasing, which would enhance the SOC and consequently the DMI, the behavior of $d^{tot}$ is not dominated anymore by the $d_{x^2-y^2}$ and $d_{xy}$ since they lie well below the Fermi level. From this aspect, chemical composition determining Fermi energy is a crucial variable for the DMI as well as crystal lattice strain. We anticipate that this study will influence future discussions on the relation between crystal structure and chemical composition of chiral thin films and microscopic origin of DMI.

## Summary

We investigated the influence of lattice strain on Dzyaloshinskii–Moriya interaction at Co/Pt interfaces. We were able to control the interaction by altering c/a ratio of the crystal structure. Also, the DMI was enhanced up to 32% by a small tensile strain of 7%. The relation between the DMI and interlayer distances of the lattice was identified by chirality-dependent energy difference of individual layers calculations. The electronic origin of enhanced-DMI was attributed to the intersection of occupied and unoccupied energy levels indicating an orbital hybridization at a certain c/a ratio. Our results represent a rational basis for controlling the DMI by suitable substrates or superlattice blocks providing the desired c/a ratio.

## Data Availability

The datasets generated and analysed during the current study are available from the corresponding author on reasonable request.
